# ENA-001 Reverses Xylazine/Fentanyl Combination-Induced Respiratory Depression in Rats: A Qualitative Pilot Study

**DOI:** 10.7759/cureus.74826

**Published:** 2024-11-30

**Authors:** Thomas L Miller, Jeanette Mathews, George C Dungan, Joseph V Pergolizzi, Robert B Raffa

**Affiliations:** 1 Clinical Development, Enalare Therapeutics, Princeton, USA; 2 Research and Development, Enalare Therapeutics, Princeton, USA

**Keywords:** bk channels, ena-001, fentanyl, rats, respiratory depression, xylazine

## Abstract

Xylazine exacerbates the respiratory depression induced by fentanyl. Because xylazine is a non-opioid, it is resistant to reversal by opioid receptor antagonists such as naloxone (e.g., Narcan^®^), thereby complicating attempts at treatment of fentanyl overdose. Antagonists of large-conductance potassium BK (big potassium) channels (BK_Ca_) in the carotid bodies reverse drug-induced hypoxia (decreased pO_2_) and hypercapnia (increased pCO_2_). In animals and human volunteers, the selective BK antagonist ENA-001 reverses the respiratory depression induced by opioids and non-opioids, i.e., it is an "agnostic" respiratory reversal agent. Given the seriousness of xylazine plus fentanyl combination (XFC) overdose, the present pilot study in rats was designed to evaluate the potential of a single intravenous bolus of ENA-001 to mitigate the acute respiratory depression induced by a prior intravenous bolus infusion of an XFC. XFC-induced respiratory depression was manifested as a decrease in pO_2_ and an increase in pCO_2_. ENA-001, but not the vehicle, rapidly reversed these XFC-induced changes. Based on the results of this pilot study, the "agnostic" nature of ENA-001 respiratory stimulation appears to extend to XFC-induced overdose. Given the urgent clinical need, additional study seems warranted.

## Introduction

In recent years, the opioid crisis has taken an even deadlier turn with the increasing use of a combination of the veterinary tranquilizer xylazine with fentanyl [1,2]. According to the Drug Enforcement Administration (DEA), xylazine was detected in more than 25% of fentanyl-related overdose deaths in some parts of the U.S. in 2021, with numbers continuing to rise [3,4]. The combination ("tranq dope") has raised serious overdose concerns for two reasons: (i) its lethal potential and (ii) its resistance to standard overdose treatments [5]. While the synthetic opioid fentanyl alone is already a leading cause of drug overdose deaths, the addition of xylazine exacerbates the risk by introducing complex challenges [6-8].

Xylazine is a non-opioid used in veterinary medicine as an animal tranquilizer, but it is not approved for use in humans [9]. It is an alpha2-adrenoceptor agonist, which depresses the central nervous system, producing sedation and muscle relaxation [10]. Xylazine is not approved for human use because it causes adverse cardiovascular effects and respiratory depression [11]. However, it is increasingly found mixed with illicit drugs, particularly fentanyl and heroin [12]. Although the exact nature of its appeal is not known, its sedative effects might intensify or prolong the euphoric effects produced by opioids [13].

One of the most significant challenges of an overdose with a xylazine plus fentanyl combination (XFC) is the limited effectiveness of opioid receptor antagonists such as naloxone [14]. Because xylazine is not an opioid, naloxone does not counter its sedative or respiratory-depressive effects [15]. The presence of xylazine in fentanyl-related overdoses is associated with increased mortality rates because it complicates the typical overdose-reversal protocol [14].

ENA-001 is a selective antagonist of large-conductance potassium BK (big potassium) channels (BK_Ca_), which are located within the carotid bodies [16,17]. In animals and human volunteers, ENA-001 reverses the respiratory depression induced by opioids and non-opioids, i.e., it is an "agnostic" respiratory reversal agent [18,19]. The objective of this study was to evaluate the potential of a single intravenous bolus of ENA-001 to reverse the augmented respiratory depression of acute intravenous infusion of XFC to rats.

## Materials and methods

Xylazine, fentanyl, and vehicle/diluent (saline) were obtained from commercial sources. ENA-001 (2-N,O-dimethylhydroxylamino-4,6-bispropylamino-s-triazine) was supplied by Enalare Therapeutics (Princeton, NJ). Doses were prepared on the day of dosing. Fentanyl and xylazine were prepared in a single solution delivered as a single intravenous bolus infusion of 2.5 mL/kg containing 20 µg/kg fentanyl and 3 mg/kg xylazine. For the formulation, 0.8 mL fentanyl citrate (50 µg/mL stock) was combined with 0.3 mL xylazine hydrochloride (20 mg/mL stock) and diluted with 3.9 mL of saline to make 5 mL of cocktail solution (1.2 mg/mL xylazine, 8 µg/mL fentanyl) delivered as 2.5 mL/kg. ENA-001 was delivered as an intravenous bolus infusion of 2.5 mL/kg containing 3.0 mg/kg ENA-001. For the formulation, 0.5 mL ENA-001 stock solution (10 mg/mL stock) was diluted to 1.2 mg/mL by adding 4.17 mL of saline. The final prepared dose formulation was adjusted to a pH of 4.1 ± 0.2 and filtered.

**Figure 1:**
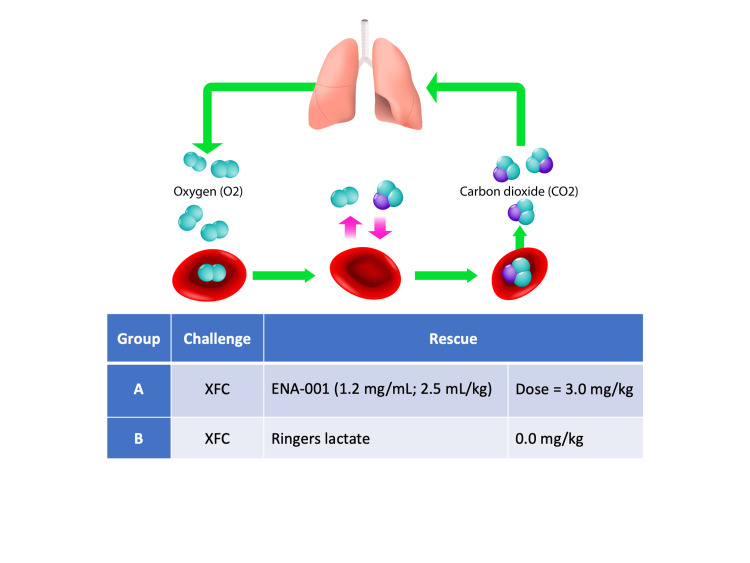
Experimental design XFC was administered by slow bolus intravenous infusion (≤15-s push) into the tail vein at Time = 0 min, followed by catheter flush with saline. ENA-001 or vehicle (Ringers lactate) was administered by intravenous infusion into the tail vein at Time = 5 min, followed by catheter flush with saline. XFC = xylazine (1.2 mg/mL; 3.0 mg/kg) plus fentanyl (8.0 µg/mL; 20 µg/kg). N = 5 per group. Image credit: licensed from iStock.com/ttsz. XFC, xylazine plus fentanyl

Male Sprague Dawley rats (approximately nine weeks of age, 241 - 373 g) were obtained from Charles River Laboratories, Inc. (Wilmington, NC). They were surgically implanted with cannulas, and then acclimated for seven to eight days before dosing in individual polycarbonate "shoebox" caging with absorbent bedding. Food (Harlan Teklad Rodent Diet #2018C) and water were available ad libitum. Conditions: room temperature approximately 20 - 26°C (68 - 79°F), relative humidity approximately 30 - 70%, a minimum of 10 air changes per hour, and a 12-h light/12-h dark cycle. All housing and handling procedures involving animals were in accordance with the Guide for the Care and Use of Laboratory Animals (The Animal Care and Use Program (ACUP) number was 230204). The number of animals used in the study was the minimum necessary for scientific validity (N = 5 per group), and the animals were euthanized by CO_2_ asphyxiation. To the knowledge of the authors, the conduct of this study resulted in no unnecessary duplication of existing data with regard to test species, test article, dose(s), route, or duration of administration. The rats were randomly allocated into two groups: (A) XFC challenge followed by ENA-001 rescue and (B) XFC challenge followed by saline control. The doses of the XFC were based on previous studies involving respiratory depressant effects of fentanyl in rats and published literature on the doses of xylazine that produce respiratory depression in rats. The dose of ENA-001 was based on a dose that produces respiratory stimulation in rats. The infusions of XFC and ENA-001 were stagger-started over an approximate one-hour period to facilitate study procedures. The experimental design is summarized in Figure 1.

## Results

The XFC induced tail rigidity in the rats immediately upon infusion (which was initiated at T = 0), and respiratory depression, as assessed by blood gas parameters, starting at the first measurement (T = 4 min), persisting to subsequent measurements (T = 10, 15, and 30 min). Respiratory depression was primarily reflected by notable decreases in pO_2_ and increases in pCO_2_. The greatest respiratory depression was observed at the T = 4 min measurement, with gradual recovery thereafter, approaching recovery to baseline values by the T = 30 min time point. Because no measurements were made prior to the 4 min measurement, it is not known if the greatest respiratory depression occurred between T = 0 and 4 min.

**Figure 2:**
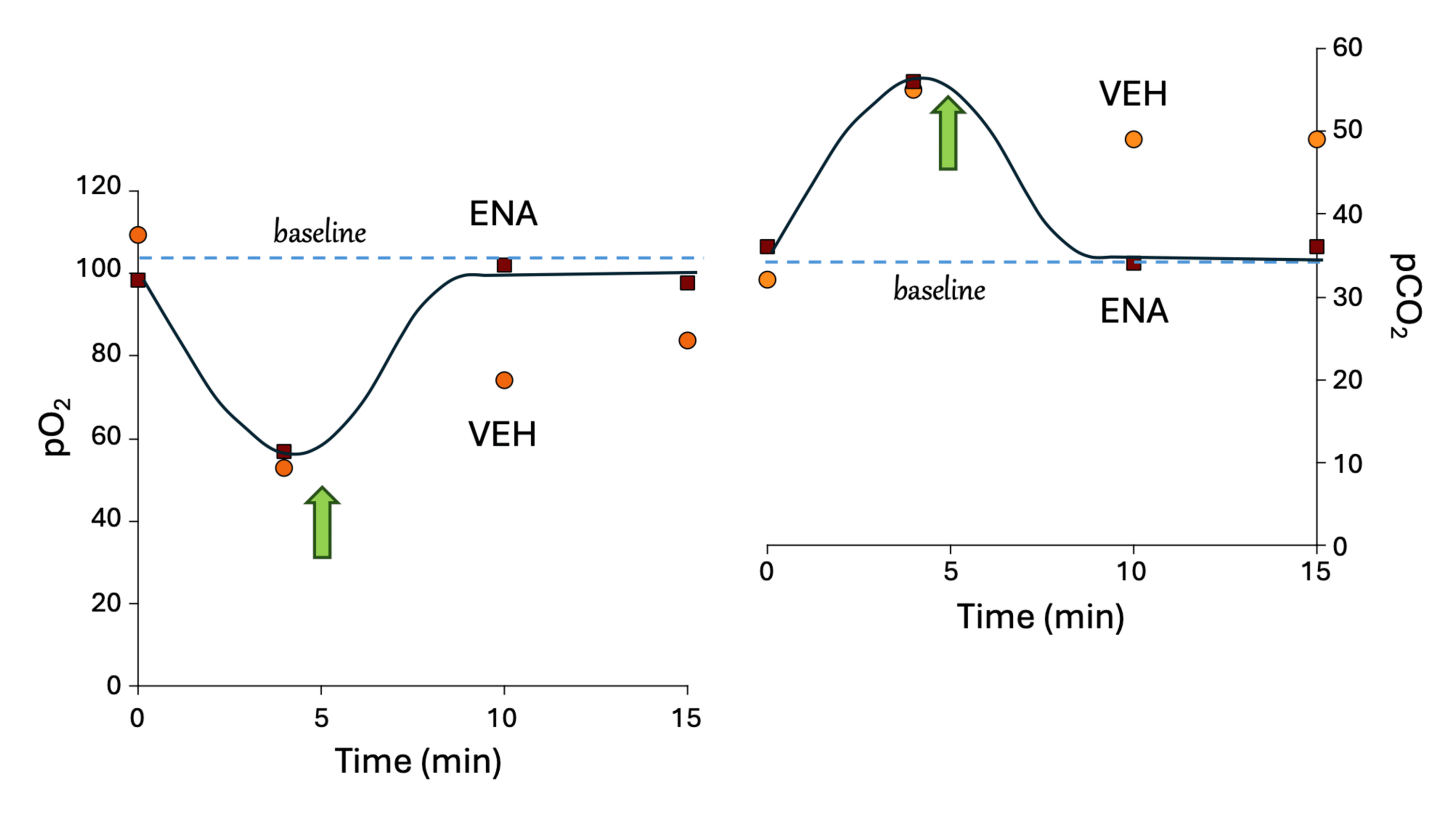
Reversal of XFC respiratory depression by ENA-001 Bolus intravenous infusion of XFC was initiated at Time = 0, and ENA-001 and vehicle were administered by bolus intravenous infusion 5 min later. Respiratory depression was measured by decreases in oxygen (pO_2_) and increases in carbon dioxide (pCO_2_). XFC, xylazine plus fentanyl combination

ENA-001, infused as a rescue bolus at T = 5 min after infusion of XFC attenuated XFC-induced respiratory depression in rats, noted at 5 minutes after the rescue bolus was administered (i.e., at the T = 10-min measurement) and persisting at the T = 15 min time point. Because no measurements were made between the 5 min and 10 min measurements, it is not known if more rapid attenuation of respiratory depression occurred between T = 5 and 10 min. The time course of respiratory depression parame was similar for pO_2_ and pCO_2_ (Figure 2).

## Discussion

An XFC creates a particularly unpredictable and dangerous drug mixture [20,21]. Both fentanyl and xylazine are central nervous system depressants that diminish brain activity, including in structures related to breathing [22]. When taken together, the combined effects can result in exacerbated respiratory depression (preprint), the leading cause of opioid overdose deaths [23]. Xylazine’s effects on muscle relaxation and sedation can exacerbate the respiratory suppression caused by fentanyl [21,24]. While fentanyl induces respiratory depression by acting on opioid receptors in the brainstem, xylazine depresses the central nervous system through its independent action on alpha2-adrenoceptors, compounding the effects and leading to a higher risk of fatal overdose [21,25]. Because xylazine is a non-opioid, it adds an additional component of risk to fentanyl overdose, because as a non-opioid, xylazine is resistant to reversal by opioid receptor antagonists such as naloxone (e.g., Narcan®). Combined intravenous injection of xylazine with fentanyl thus complicates attempts at treatment of fentanyl overdose [26]. The present study suggests that a selective BK channel antagonist might reverse respiratory depression induced by the combination. A limitation of such a pilot study, however, is the small number of animals and lack of numerical values or statistical measures (e.g., mean ± SEM for pO_2_ and pCO_2_, percentage changes, or p-values), which limits the ability to assess the magnitude and significance of the effects for the clinical setting.

## Conclusions

Since the combination of xylazine with fentanyl is a difficult societal and medical challenge, the availability of new pharmacotherapeutic options seems desirable. The present pilot study in rats was designed to evaluate the potential of a single intravenous bolus of an investigational BK channel antagonist in clinical development (ENA-001) to mitigate the acute respiratory depression induced by prior intravenous bolus infusion of an XFC to rats. ENA-001, but not vehicle, reversed XFC-induced changes (decrease in pO_2_ and increase in pCO_2_) within 5 minutes and possibly sooner. Based on the results of this pilot study, the "agnostic" nature of ENA-001 appears to extend to XFC-induced respiratory depression, suggesting that additional study of the clinical implications is warranted.
